# Understanding Early Risk Factors of Preschool Disruptive Behaviors in a Population-Based Birth Cohort: Why Does Comorbidity Matter?

**DOI:** 10.3390/healthcare12232380

**Published:** 2024-11-27

**Authors:** Rene Carbonneau, Frank Vitaro, Mara Brendgen, Michel Boivin, Richard E. Tremblay

**Affiliations:** 1 Department of Pediatrics, University of Montréal, Montréal, QC H3T 1J7, Canada; 2 Centre de Recherche Azrieli du CHU Sainte-Justine, Montréal, QC H3T 1C5, Canada; 3 Research Unit on Children’s Psychosocial Maladjustment, University of Montréal, Montréal, QC H3T 1C5, Canada; 4 Department of Psychoeducation, University of Montréal, Montréal, QC H3C 3J7, Canada; 5 Department of Psychology, University of Québec in Montréal, Montréal, QC H3C 3P8, Canada; 6 Department of Psychology, Université Laval, Québec City, QC G1V 0A6, Canada; 7 Department of Psychology, University of Montréal, Montréal, QC H3C 3J7, Canada

**Keywords:** comorbidity, disruptive behaviors, externalizing, trajectories, preschool years, risk factors

## Abstract

Background/Objectives: Research on early risk factors for disruptive behaviors (DBs: hyperactivity–impulsivity/HI, non-compliance/NC, or physical aggression/PA) has predominantly focused on individual DBs in silos (i.e., HI, NC, *or* PA) or the broader category of externalizing, reporting mostly common risk factors among them. However, studies addressing DB comorbidity, i.e., the simultaneous occurrence of more than one DB, showed differences in risk factors among DB comorbid profiles. Aiming to clarify this discrepancy, the present study compared the early risk factors associated with different longitudinal patterns (i.e., trajectories) of single-DBs (HI, NC, PA) with risk factors associated with monomorbid (HI_only_, NC_only_, PA_only_) and comorbid (HI + NC, NC + PA, HI + NC + PA) joint-DBs trajectories during the preschool period. Methods: In a population-based birth cohort (N = 2045), parents’ pre-conception characteristics, pregnancy and perinatal conditions, and age 5 months child and family characteristics were used to compare children following single-DB and joint-DBs high trajectories to children following low or moderate trajectories. The DB trajectories were derived from mother ratings at ages 1½, 2½, 3½, 4½, and 5 years. Results: More risk factors were identified for single-DB high trajectories than for joint-DBs high trajectories. On average, children on a single-DB high trajectory shared only 44.2% of their risk factors with children on a related joint-DBs high trajectory. Moreover, high trajectories of single-DBs shared a larger proportion of their risk factors than did high trajectories of joint-DBs. The findings show that categories of DBs include different subgroups of children based on their comorbidity patterns across DBs, which are differentially linked to early risk factors. Conclusions: Addressing comorbidity when investigating early risk factors of preschool DBs may improve our understanding of the etiological processes leading to these distinct but related behaviors and increase our ability to intervene upstream to prevent the earliest forms of potentially life-altering psychopathological conditions.

## 1. Introduction

Disruptive behaviors (DBs) in children are linked to significant academic, social, professional, as well as physical and mental health challenges from early schooling through adolescence [1,2,3] and into early adulthood [4,5], potentially impacting an individual’s entire life [6,7,8]. Recent studies indicate that DBs can manifest as early as the preschool years [1,9,10,11]. From a developmental psychopathology perspective, elucidating the developmental conditions associated with DBs and preventing their onset necessitate a thorough understanding of their origins and progression [12,13,14]. To that end, the identification of fine-grained individual behaviors that usually fall under the DBs umbrella (i.e., hyperactivity–impulsivity, non-compliance–opposition, or physical aggression) is deemed essential [15]. A related key strategy is to explore the comorbidity between these specific DBs from their earliest manifestations onward and determine whether risk factors present in children’s early years are common or specific to each of them [16]. Clarifying developmental models that incorporate individual and socio-familial risk factors, potentially distinguishing between DBs [17], is the first step to early, personalized preventive interventions [14]. Given that early childhood is characterized by rapid maturation and behavioral changes [14,16], longitudinal studies are required to accurately track the progression of DBs, avoiding the limitations of cross-sectional assessments. Employing both variable- and person-centered approaches enables researchers to describe these behaviors over time and compare a child’s behavior with that of age-matched peers [18,19].

Recent developmental models of psychopathology emphasize a common externalizing factor among DBs and across time that could explain their developmental comorbidity—the simultaneous occurrence of two or more problem behaviors [4,20]—and unique environmental factors that independently influence individual DBs, supporting the idea of partially common etiologies [21,22]. To date, research on early risk factors for DBs in preschoolers has predominantly focused on either individual DBs or the broader category of externalizing behaviors. Commonly identified risk factors include prematurity or low birth weight for hyperactivity–impulsivity, sibling presence for physical aggression, and a host of factors similar across DB categories, such as poverty, low socioeconomic status (SES), parental education, maternal smoking during pregnancy, younger maternal age, parental psychopathology (especially conduct problems and depression), harsh or coercive parenting, and family dysfunction [23,24,25,26,27,28,29,30,31,32,33]. These risk factors are also associated with broader externalizing behaviors in preschool children [34,35,36]. Thus, based on the above results, early risk factors associated with different types of DBs are largely similar.

Yet, the rare studies examining risk factors for longitudinal DB patterns in preschool children while taking into account comorbidity reported significant differences in risk factors linked with different comorbid profiles of DBs [17,31]. These results are seemingly inconsistent with those from the studies of individual DBs noted above, which mostly reported similar risk factors across different types of DBs. Clarifying the discrepancy between these findings is an important etiological issue with potential benefits for early childcare and preventive intervention. To address this issue in children’s early years, a methodological strategy should meet two criteria. First, a comparative examination should be conducted, within the same sample, of the association of early risk factors with (a) different types of DBs and (b) comorbidity patterns among these DBs. Second, because early childhood is a period of rapid change and maturation, a longitudinal assessment of DBs is essential to avoid the caveats of cross-sectional studies. To this end, a strategy employing a dual variable- and person-centered approach allows for tracking the progression of DBs in each child compared to same-age peers. The identified longitudinal patterns (i.e., trajectories) represent a comprehensive basis for developmental models that examine specific or common risk factors among DBs.

### The Present Study

**Figure 1:**
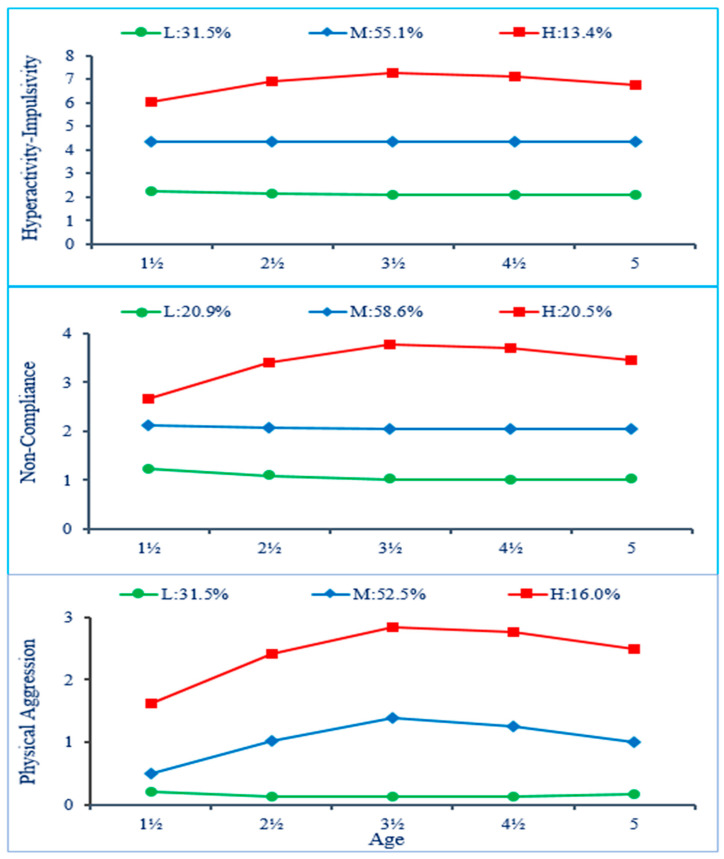
Trajectories of single-DBs in preschool years. Frequencies for each DB rating scale are displayed. Trajectories: Low (L), Moderate (M), High (H). Adapted from [1]. Copyright 2016 by Creative Commons License Attribution 4.0 International. Reprinted with permission.

**Figure 2:**
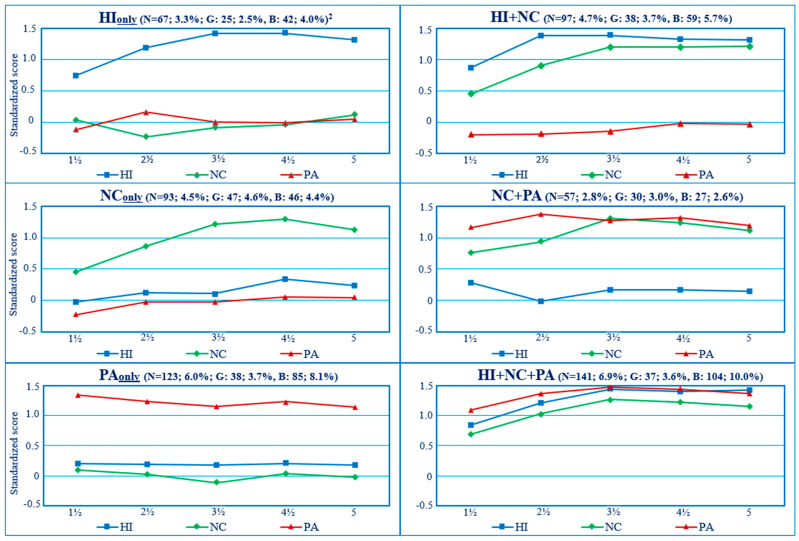
Joint-DBs high trajectory group’s observed means from age 1½ to 5 years ^1^. HI: hyperactivity–impulsivity; NC: non-compliance; PA: physical aggression. The co-occurrent trajectory class of HI + PA (i.e., without high NC) was not part of the observed joint trajectory classes. ^1^: Standardized scores. ^2^: % sample; Girls, Boys. Adapted from [1]. Copyright 2016 by Creative Commons License Attribution 4.0 International. Reprinted with permission.

**Figure 3:**
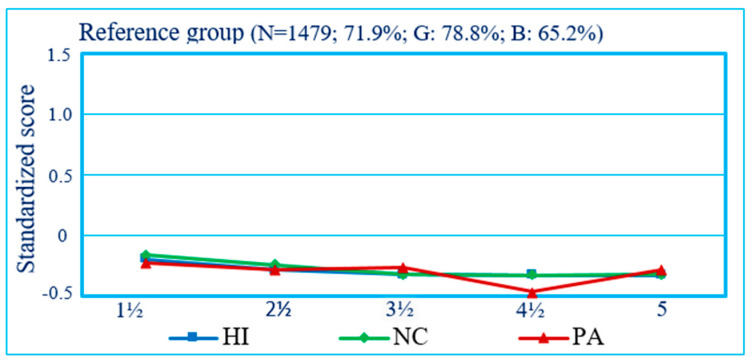
Reference group for joint-DBs high trajectory groups: Children following a low or moderate trajectory for all DBs (% sample; Girls, Boys). From 1½ to 5 years, the mean of the reference class ranged from −0.20 to −0.33 (average: −0.29) for HI, from −0.16 to −0.33 (average: −0.28) for NC, and from −0.23 to −0.28 (average: −0.31) for PA.

The present study aimed to address the above etiological issue by capitalizing on longitudinal DB trajectory groups of preschoolers identified in a previous report, which documented the joint longitudinal courses of mother-rated hyperactivity–impulsivity (HI), non-compliance (NC), and physical aggression (PA) in a representative birth cohort [1]. The results of that study showed the following: (1) for each type of DB, children followed either a *Low*-, *Moderate*-, or *High*-frequency trajectory from ages 1½ to 5 years (Figure 1); (2) the *Moderate* trajectory was the modal category (followed by the *Low* category) for all three DBs, showing the prevalence of such behaviors in preschoolers and prompting the use of the *High* trajectory as an indicator of children’s maladjustment; (3) between 13% and 20% of children manifested higher levels of either HI, NC, or PA; (4) developmental comorbidity between HI, NC, and PA was frequent, with 14.4% of the sample (boys: 18.3%; girls: 10.4%) following the highest trajectory for more than one DB (Figure 2); and, (5) in comparison to children following a *Low* or *Moderate* trajectory for all three DBs (Figure 3), those who followed a *High* trajectory for at least one type of DB received higher ratings from their teachers on indicators of DBs and school adjustment in first grade. In a second study, the risk factors during the prenatal to early postnatal period preceding the above trajectories were examined in relation to the ‘pure’ or monomorbid (i.e., high-HI_only_, NC_only_, PA_only_) and comorbid (high-HI + NC, NC + PA, HI + NC + PA) longitudinal patterns of joint-DBs throughout preschool, showing differential associations of risk factors across DB patterns [17].

The present study was concerned with comparing (across the same period and in the same population birth cohort) (a) the early risk factors linked to preschool single-DB high trajectories of different DBs (i.e., HI, NC, or PA), when contrasted with their corresponding *Low* or *Moderate* trajectories, and (b) the early risk factors linked to joint-DBs high trajectories (i.e., reflecting the parallel course of all three DBs simultaneously), which included one or more high trajectories among these DBs (i.e., high-HI_only_, high-NC_only_, high-PA_only_, high-HI + high-NC, high-NC + high-PA, high-HI + high-NC + high-PA), when contrasted with *Low* or *Moderate* trajectories for all three DBs (Figure 3). Thus, joint trajectory groups represent subgroups of a corresponding single-DB high trajectory that also reflect children’s course for the other types of DBs. For example, the single-DB high trajectory group for HI includes children who are high on HI but low or moderate on NC and PA (i.e., high-HI_only_), children who are high on HI and high on NC but low or moderate on PA (i.e., high-HI + NC), and children who are high on all three DBs (i.e., high-HI + NC + PA; see Section 2.2.1 for further details). Adopting a longitudinal, developmental psychopathology perspective and based on previous studies examining early risk factors of DBs [23,24,25,26,27,28,29,30,31,32,33,34,35,36], risk factors were investigated across four time periods [17]: (1) Parents’ pre-conception characteristics (i.e., parents’ conduct problems when they were adolescents, parents’ education, and mother’s age at the birth of her first child); (2) pregnancy characteristics (i.e., mother’s use of tobacco, alcohol, and drugs during pregnancy); (3) perinatal characteristics (i.e., parents’ age, child’s sex, prematurity and birthweight, number of siblings); (4) age 5 months child and family environment characteristics (i.e., child’s temperament, family structure, SES, family dysfunction, parents’ psychosocial adjustment, and parenting practices).

Given the number of potential risk factors stemming from previous investigations of early longitudinal patterns of DBs noted earlier and, among the latter, the paucity of studies that have taken into account the comorbidity among DBs, the present study remains exploratory with regard to the specific nature and strength of the risk factors that may be identified through the comparison of their association with single-DB and joint-DBs trajectory groups. However, based on developmental models of psychopathology and the empirical reports described above, we expected that, when comparing children following a high trajectory to children following a low or moderate trajectory of DBs, (1) a core of risk factors would be common to single-DB high trajectory groups (i.e., high-HI; high-NC; high-PA) and to joint-DBs high trajectory groups (i.e., including one or more high trajectory among DBs); (2) single-DB high trajectory groups should have more risk factors and share a higher proportion of them than the more refined joint-DBs high trajectory groups; (3) risk factors associated with a given single-DB high trajectory group (e.g., high-HI) are likely to be associated with its subcategories represented by joint-DBs high trajectory groups, including the same high trajectory (i.e., high-HI_only_, HI + NC, HI + NC + PA); and (4) risk factors common to multiple single-DB high trajectories (i.e., high-HI, high-NC, or high-PA) should have the strongest association with comorbid joint-DBs high trajectory groups (i.e., high-HI + NC, NC + PA, HI + NC + PA).

## 2. Materials and Methods

### 2.1. Participants

The participants originated from a birth cohort of 2045 infants (50.3% boys) representative of the children born (single births) in the province of Quebec, Canada, in 1997–1998 (Québec Longitudinal Study of Child Development; QLSCD) [37]. Children and their family were assessed 5 months after birth (T1) and yearly afterwards. The assessments were based on a face-to-face interview with the most knowledgeable person about the child (average duration: 45 min). In addition, self-report questionnaires were administered to both parents. Assessments at the ages of 1½, 2½, 3½, 4½, and 5 years (T2-T6; average response rate of 94.8%) were used to identify the trajectories of DBs [1]. The latent class growth mixture method used [18,19] accommodates missing data, so that participants with incomplete assessments across repeated measures could be included, and all values available at each time used for the trajectory estimation (N = 2045). The risk factor variables used in the present study were assessed when the child was aged 5 months (T1) and showed a low proportion of missing values (3.9% overall; 0–3.7% for 80.6% of variables). Six predictors (19.4%) had, on average, 11.9% of missing values (range: 7.9–16.1%) and were all paternal psychosocial characteristics. Based on the overall proportion of missing data and the conditional missingness pattern in relation to paternal information, data were considered missing at random (MAR). In such cases, multiple imputation techniques are appropriate [38,39]. Multiple imputation from linear and logistic regressions using all variables in this study was thus applied to impute the missing values.

### 2.2. Measures

#### 2.2.1. Trajectories for Different Single DBs and Joint Trajectories Across DBs During the Preschool Years

Preschool trajectories of HI, NC, and PA, based on maternal ratings, were generated as a first step of analysis using Group-Based Trajectory Modeling in SAS [19,40]. The trajectories obtained for single DBs are shown in Figure 1. The joint trajectories were estimated in a second step through a procedure analyzing the trajectories’ joint and conditional probabilities across DBs [19] to describe their co-occurrence (i.e., parallel course) from age 1½ through 5 years. Figure 2 shows the observed standardized means of HI, NC, and PA for the joint-DBs high trajectory groups. As shown, children following a joint-DBs high trajectory for only one DB (i.e., monomorbid) also followed a low or moderate trajectory for the other DBs. For example, high-HI_only_ stands for high HI + low or moderate NC + low or moderate PA and is designated as HI_only_ for simplicity. Similarly, children following a joint-DBs high trajectory for two DBs (i.e., comorbid) also followed a low or moderate trajectory for the remaining third DB. For example, high-NC + PA stands for high NC + high PA + low or moderate HI and is designated as NC + PA for simplicity. Finally, a joint-DBs high trajectory for all three DBs is designated as HI + NC + PA for simplicity. The proportion of boys and girls following a joint-DBs trajectory including at least one *High* trajectory for a given DB was, respectively, 34.8% and 21.3%. It is noteworthy that the comorbidity profile of high HI + PA (i.e., high HI + high PA + either low or moderate NC) was not observed among the trajectory groups, suggesting that a high level of HI and PA likely comes with a high level of NC in preschoolers. Among children following a high trajectory for a single-DB (HI, NC, *or* PA), the corresponding comorbid joint-DBs high trajectory groups (i.e., HI + NC, NC + PA or HI + NC + PA) represented the majority of children for both genders (i.e., 63.8% to 74.7% of girls and 60.9% to 80.6% of boys).

#### 2.2.2. Risk Factors

Most (25/31) risk factors used in the present study originated from our previous report examining risk factors of the ‘pure’ or monomorbid and comorbid longitudinal patterns of DBs throughout preschool [17]. However, 6 risk factors were added to examine parents’ education and alcohol and drug use and the family’s formal status with regard to poverty, and analyses for the present study were based on this new, more inclusive and discriminant set of risk factors regarding these latter aspects. *Parents’ early characteristics* were based on two specific measures for each parent: (1) a score on a rating scale including five different conduct problems presented prior to finishing high school and referring to DSM-IV criteria for conduct disorder and antisocial personality disorder (alpha: 0.90, mother; 0.80, father) [41]; (2) an indicator of education based on whether each parent was holding a high school degree or not. Mother’s age at the birth of her first child (not necessarily the participating target child) was also included among these early background factors. *Prenatal factors* consisted of binary indicators (yes/no) regarding maternal use of tobacco (at least 1 cigarette per day or not), alcohol (at least once per week or not), or illegal drugs (any) during pregnancy. *Perinatal factors* comprised the following information: parents’ age at the target child’s birth, child’s gender, premature birth (i.e., gestational age of less than 37 weeks) or low birthweight (<2.5 kg), and number of siblings.

At age 5 months, the following *child and family environment characteristics* were assessed: (1) child’s difficult temperament based on both parents’ ratings of seven items from the difficult temperament scale of the Infant Characteristics Questionnaire (ICQ; alpha: 0.77, mother; 0.79, father) [42]; (2) family structure, defined as intact when both biological parents were living together or otherwise as non-intact; (3) family dysfunction (e.g., with regard to communication, affection, control, problem resolution) based on 12 mother-rated items of the McMaster Family Assessment Device (alpha: 0.88) [43]; (4) an indicator of whether family income was below Statistics Canada’s low income cut-off in the past year; (5) each parent’s depressive symptoms measured with the abbreviated version (12 items) of the Center for Epidemiologic Studies Depression Scale (CES-D; alpha: 0.78, mother; 0.73, father) [44]; (6) a similar rating scale of conduct problems described above but in reference to the post-high-school/adult period (alpha: 0.85, mother; 0.87, father); (7) each parent’s self-reported alcohol use (frequency—last 12 months, 0–7: 1 < once/month; 4 = once/week; 7 = every day) and illicit drug use (0 = none, 1 = 1, and 2 = 2^+^ types of drugs); (8) measures of both mother’s and father’s parenting practices based on the Parental Cognition and Conduct Toward the Infant Scale [45] and describing parents’ perceptions of affection and pleasure in parent–child interactions, self-efficacy, coercion, overprotection, as well as mother–child constructive interactions (e.g., frequency of play and support). Cronbach’s alphas ranged between 0.69 and 0.79 for the different parenting scales.

### 2.3. Data Analysis

Risk factors related to parents’ early characteristics and to the prenatal, perinatal, and age 5-month periods were analyzed using logistic regression (LR) based on a generalized linear model (hereafter: GLM) platform. GLM provides a unified approach to modelling for all types of response variables within the exponential family of distributions, including normal, Poisson, and binomial distributions [46]. In line with the strategy used in previous studies [1,17,23,24,25,26,27,28,29,30,31,32,33,34,35,36], for each single DB, children following a *High* trajectory (high-HI, NC, or PA; i.e., from the first step of the trajectory analyses) were compared to their peers following *Low* or *Moderate* trajectories. Children in joint-DBs high trajectory groups—whether in monomorbid (i.e., *high*-HI_only_, NC_only_, and PA_only_,) or comorbid (*high*-HI+NC, NC + PA, HI + NC + PA) trajectory groups (i.e., from the second step of the trajectory analyses)—were compared to a reference group aggregating their peers who followed *Low* or *Moderate* trajectories for all three DBs across the preschool years [1]. This decision was made to secure risk factors specific to high trajectories without multiplying the number of comparisons and given that moderate, followed by low trajectories, represented large groups suggesting normative levels of DBs in preschoolers (on average, 55.4% and 27.8%, respectively, across the three DBs). A series of preliminary bivariate analyses were conducted to test whether the associations between each risk factor (i.e., predictor) and the trajectory groups met a minimal significance criterion (*p* < 0.25) for predictors to be included in the multivariate analyses [47]. All predictors met this criterion, with the exception of father’s self-efficacy and overprotection, which were excluded from subsequent analyses. Bivariate associations tested between the thirty-one predictors (all coefficients < 0.47) remained within acceptable levels of collinearity [48]. Continuous variables were standardized, and group members’ posterior probabilities of assignment in trajectories were used as sampling weights in LR in order to account for uncertainty in the assignment of individuals to trajectory groups [18,19].

Multivariate LR analyses with a backward elimination approach were used to identify the optimal combination of risk factors associated with single-DB high trajectories and joint-DBs high trajectory groups. GLM uses an information theoretic approach, which is considered a more adequate tool than null hypothesis statistical testing when model fitting involves variable selection procedures [49,50]. In particular, GLM’s information criteria (IC) include penalties for the number of model parameters, which leads to adjusted estimates, whereas NHST-based stepwise regression procedures have been criticized to have an inherent multiple testing problem and elevated Type I error rates [49]. The full risk model including all candidate predictors was fit first in order to examine the predictors’ contribution within the multivariate context and to provide IC initial fit statistics. Akaike information criterion (AIC) and Bayesian information criterion (BIC) statistics were examined. For all IC, the smallest value indicates the best model. Models with IC differences of less than 2 provide little evidence in support of one specific model, whereas a difference of 10 or more is considered strong evidence [51]. Monitoring fit statistics variations and estimates of the predictors’ coefficients and significance levels as indicators of their relative contribution, the model was sequentially reduced to the most parsimonious combination of risk factors that provided the best fit to the data [47,49] while differentiating each DB high trajectory group from the reference group. Finally, the results with single-DB high trajectory groups and joint-DBs high trajectory groups were compared to identify common and specific risk factors. When different trajectory groups shared a risk factor, the overlap between confidence intervals was examined, and LR analyses including covariates were conducted to test whether the difference in the strength of association between trajectory groups was statistically significant.

## 3. Results

### 3.1. Risk Factors for Single-DB High Trajectory Groups

The model selection procedure with single DBs examined separately in the first step of the predictive analyses started with a full model including all predictors (Table 1). For all three types of DBs, the final model provided a better fit than the initial full model that included all predictors, with IC differences ranging between −26.125 and −32.633 for AIC and between −143.617 and −161.315 for BIC, indicating a strong improvement in fit (Table 2). The selection process identified ten risk factors associated with the single-DB *High*-HI trajectory group, eight with the *High*-NC trajectory group, and eight with the *High*-PA trajectory group. Over a total of 14 statistically significant risk factors, (1) four (28.6%) were common to all single DBs: father’s adolescent conduct problems (CP), mother’s younger age at child’s birth and depression, and male gender; (2) four were common to two single DBs: non-intact family and mother’s coercion (HI; NC), mother’s smoking during pregnancy (HI; PA), and mother’s adult CP (NC; PA); and (3) six (42.9%) were specific to one single DB or another: prematurity or low birthweight, father’s depression and mother’s overprotection (HI), father’s coercion (NC), number of siblings, and family dysfunction (PA).

### 3.2. Risk Factors for Joint-DBs High Trajectory Groups

The model selection procedure with all six joint-DBs high trajectory groups showed that, compared to the initial full model that included all predictors (Table 3), the final model (Table 4) provided a better fit, with IC differences ranging between −23.759 and −38.422 for AIC and between −156.937 and −187.545 for BIC, indicating a strong improvement in fit. Six risk factors were associated with the joint-DBs high trajectory group of HI_only_ (Table 4). Two, male gender and mother’s depression, were shared with the other joint-DBs high trajectory groups including HI, and four were specific to this group: mother’s age at first child’s birth, prematurity or low birthweight, father’s depression, and mother’s overprotection.

Three risk factors were associated with the joint-DBs high trajectory group of NC_only_. One was shared with the joint-DBs high trajectory group of HI + NC + PA, mother’s age at child’s birth, and two were specific factors, mother’s frequency of alcohol use and coercion. Five risk factors were associated with the high trajectory group of PA_only_. Four were shared with other joint-DBs trajectory groups including PA, family dysfunction, mother’s adult CP, number of siblings, and male gender, and one was specific to the trajectory group of PA_only_, father’s adult CP. The comorbid joint-DBs high trajectory group of HI + NC had six statistically significant risk factors. Three were shared by joint-DBs trajectory groups with a common high DB trajectory, male gender, mother’s depression, and mother adult CP, and three were specific to the trajectory group of HI + NC, lower number of siblings, income below poverty level, and child’s difficult temperament. The trajectory group of NC + PA had six risk factors. Three were shared with joint-DBs trajectory groups with a common high DB trajectory, mother’s adult CP, a higher number of siblings, and family dysfunction, and three were specific risk factors to the trajectory group of NC + PA, father’s younger age at child’s birth, a lower likelihood to have an income below poverty level, and father’s coercion. Finally, the comorbid joint-DBs trajectory group of HI + NC + PA had six risk factors. Four were shared with other joint-DBs trajectory groups, mother’s younger age at child’s birth, male gender, a higher number of siblings, and mother’s depression, and two were specific factors, father’s adolescent CP and mother’s smoking during pregnancy.

### 3.3. Comparison of Risk Factors for Single-DB and Joint-DBs High Trajectory Groups

When comparing statistically significant risk factors associated with single-DB high trajectory groups with those associated with joint-DBs high trajectory groups, male gender and maternal depression were the most common risk factors, shared by all single-DB high trajectory groups, by monomorbid joint-DBs high trajectory group of HI_only_, and by the comorbid joint-DBs high trajectory groups of HI + NC and HI + NC + PA. Male gender was also linked with the high trajectory group of PA_only_.

**Figure 4:**
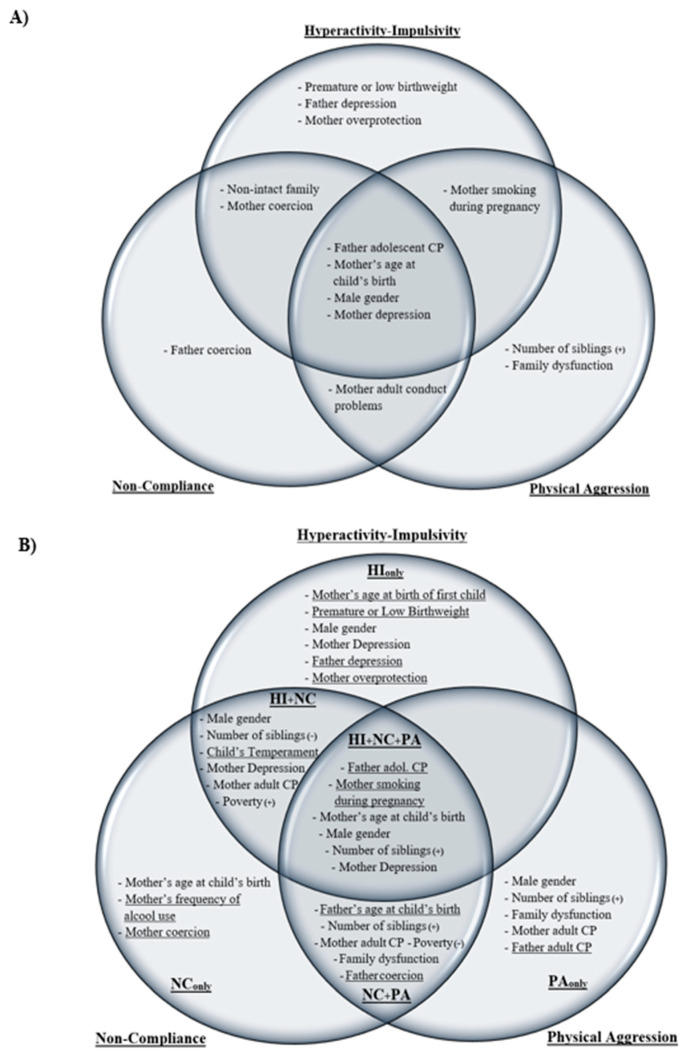
Risk factors associated with single-DB high trajectory groups (**A**) and joint-DBs high trajectory groups across DBs (**B**). Risk factors specific to trajectory groups are underlined in (**B**).

Taking an overall perspective on the number of risk factors, the three single-DB high trajectory groups were associated with a total of 14 risk factors. Six (42.9%) factors were specific to one single-DB high trajectory group or another, and eight (57.1%) were shared risk factors across single-DB high trajectories (Figure 4A). In comparison, the six joint-DBs high trajectory groups cumulated 19 risk factors; twelve (63.2%) were specific to one trajectory group or another, and seven (36.8%) were shared with other joint-DBs high trajectory groups (Figure 4B). Notably, the three comorbid joint-DBs high trajectory groups, which cumulated 12 different risk factors, only shared four (33.3%) of these factors, while the greater part of the risk factors (eight; 66.7%) was specific to one joint-DBs trajectory group or another, showing comorbidity among children’s DBs across preschool years.

Looking more specifically at the risk factors associated with a given high trajectory group, the following pattern emerged: On average, 77.5% of risk factors linked to a single-DB high trajectory group were shared with other single-DB high trajectory groups, and 22.5% of risk factors, on average, were specific to that single-DB trajectory. For example, out of ten risk factors observed for single-DB high-HI, seven (70%) were shared with at least one other single-DB high trajectory group (i.e., high-NC or high-PA or both) and three (30%) were specific to single-DB high-HI. In comparison, 59.4% of the risk factors, on average, associated with a joint-DBs high trajectory group were shared with at least one other joint-DBs trajectory group, and 40.6% of risk factors were specific to one joint-DBs high trajectory group or another.

Correspondence between risk factors of single-DB high trajectories and their related joint-DBs high trajectory groups showed that, on average, 40.0% of risk factors of the single-DB high trajectory group of HI corresponded to those of a specific related joint-DBs trajectory group or another (i.e., HI_only_, HI + NC, or HI + NC + PA). For NC, on average, 34.4% of the risk factors of the single-DB high trajectory group corresponded to those of a specific related joint-DBs trajectory group. For PA, this proportion was 58.3%. Two of the ten risk factors of the single-DB high trajectory group of HI, non-intact family and mother coercion, were not related to any of its corresponding joint-DBs high trajectory groups, and thus, eight factors were associated with one or more related joint-DBs high trajectory groups (Table 2 and Table 4). For NC, all risk factors but non-intact family were associated with at least one corresponding joint-DBs high trajectory group, as were all eight risk factors of the single-DB high trajectory group of PA with its related joint-DBs high trajectory groups.

Overall, five risk factors associated with joint-DBs high trajectory groups were not linked with any single-DB high trajectory group: mother’s age at birth of first child, father’s age at child’s birth, income below poverty level, father’s adult CP, and mother’s frequency of alcohol use. Additionally, five risk factors, smoking during pregnancy, number of siblings, family dysfunction, mother adult CP, and father coercion, associated with a specific joint-DBs high trajectory group were not linked with a related single-DB high trajectory group; for example, smoking during pregnancy was linked with the trajectory group of HI + NC + PA but not with the single-DB high trajectory group of NC.

**Figure 5:**
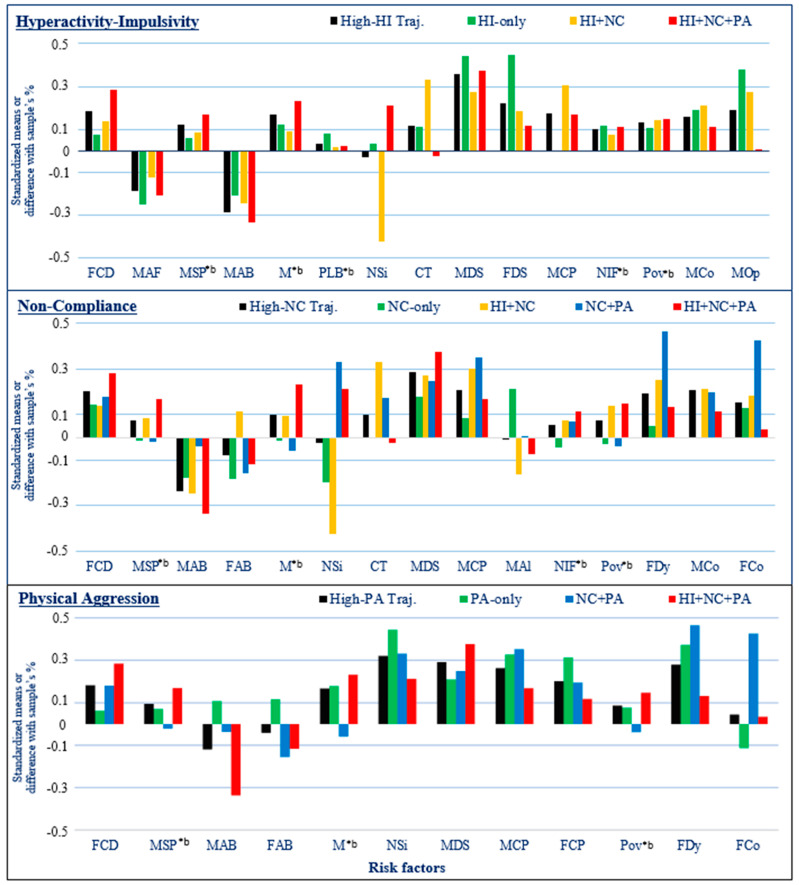
Standardized (unadjusted) means for continuous-discrete variables or difference with the sample proportion for binary variables (*^b^) for risk factors associated with each single-DB high trajectory group and with the corresponding joint-DBs high trajectory groups among HI, NC, and PA. FCD: Father’s adolescent CD; MAF: Mother’s age at birth of first child; MSP: Mother smoking during pregnancy; MAB: Mother’s age at child’s birth; FAB: Father’s age at child’s birth; M: Male; PLB: Premature or low birthweight; Nsi: Number of siblings; CT: Child’s difficult temperament; MDS: Mother’s depression symptoms; FDS: Father’s depression symptoms; MCP: Mother’s adult conduct problems; FCP: Father’s adult conduct problems; MAl: Mother’s frequency of alcohol use; NIF: Non-intact family; Pov: Poverty; FDy: Family dysfunction; MCo: Mother’s coercion; MOp: Mother’s overprotection; FCo: Father’s coercion.

The absence of commonality between some risk factors of joint-DBs high trajectory groups and a related single-DB high trajectory group is likely due to the differences in association, whether in magnitude or direction, between these factors and different joint-DBs high trajectory groups. For instance, a higher number of siblings was related to the trajectory groups of NC + PA and HI + NC + PA (OR ranging between 1.46 and 1.63), while a lower number of siblings was linked with the trajectory group of HI + NC (OR = 0.61), and no association was observed for the single-DB trajectory group of high-NC. Similarly, the children of this latter trajectory group were more likely to live in a family having an income below poverty level (OR = 1.97), while the children of the trajectory group of NC + PA were less likely to do so (OR = 0.44), and no association was observed for the single-DB trajectory group of high-NC. Figure 5 shows the means and proportions for the risk factors associated with the single-DB high trajectories of HI, NC, and PA and their related joint-DBs high trajectory groups. Differences were observed among joint-DBs trajectory groups sharing a common high DB for a number of risk factors (e.g., smoking during pregnancy-MSP, father’s age at child’s birth-FAB, number of siblings-Nsi, both parents’ adult conduct problems-MCP and FCP, mother’s frequency of alcohol use-MAl, poverty-Pov, family dysfunction-FDy, and father’s coercion-FCo). Thus, in many cases, the mean for a single-DB high trajectory group masked the differences among children in the related joint-DBs high trajectory groups.

Finally, comparing the strength of the associations between risk factors and different single-DB high trajectory groups or joint-DBs high trajectory groups suggested that they were not significantly different. The only exception was a difference in OR with regard to child’s sex (*p* = 0.028) between the HI + NC and HI + NC + PA trajectory groups (i.e., with males more represented in the latter).

## 4. Discussion

The aim of the present study was to compare the early risk factors associated with preschool high trajectories of individual single DBs (i.e., HI, NC, or PA) with risk factors linked to joint-DBs high trajectories among these three types of DBs, respectively used as longitudinal indicators for each type of DB, and their patterns of monomorbidity (i.e., HI_only_, NC_only_, PA_only_) and comorbidity (i.e., HI + NC, NC + PA, HI + NC + PA) between age 1½ and 5 years in a population birth cohort. Contrary to our first hypothesis, no risk factor was common to all single-DB high trajectory groups and to all comorbid joint-DBs high trajectory groups. Moreover, only male gender and maternal depression were common to all single-DB high trajectory groups and to two out of three comorbid joint-DBs high trajectory groups (HI + NC and HI + NC + PA). Conversely, the hypothesis of a larger number of risk factors and a higher proportion of these shared by broader single-DB high trajectories compared to more refined joint-DBs high trajectory groups was supported by the results.

On average, single-DB high trajectories shared only 44.2% of their risk factors with a related joint-DBs high trajectory group, bringing partial support to our third hypothesis. A few risk factors of single-DB high trajectories were not related to any of their related joint-DBs high trajectory groups, possibly due to reduced statistical power. Over half (10/19) of all risk factors associated with joint-DBs high trajectory groups were not linked with any (5) or with one (5) corresponding single-DB high trajectories. This lack of correspondence is likely attributable to the differences in association (for some cases, in opposite directions) between risk factors and different joint-DBs high trajectory groups related to the same single-DB high trajectory. In sum, there was some correspondence between risk factors of a single-DB high trajectory group and those of its related joint-DBs high trajectory groups, but there were also important variations across trajectory groups and, overall, fewer similarities than expected.

Interestingly, the differences in the association between risk factors and trajectory groups concerned mostly the presence/absence or the direction of this association. In contrast, the strength of the statistically significant associations observed between risk factors and single-DB or joint-DBs trajectory groups was not substantially different across trajectory groups (except for the difference regarding child gender between the trajectory groups of HI + NC and HI + NC + PA). Thus, contrary to our fourth hypothesis, risk factors common to multiple single-DB high trajectory groups did not show a stronger association with comorbid joint-DBs high trajectory groups. However, these risk factors (father’s adolescent CP, mother’s younger age at child’s birth, male gender, and mother’s depression) were associated with the largest subgroup within each single-DB high trajectory, the comorbid joint-DBs high trajectory group of HI + NC + PA. This suggests that the commonality between risk factors linked to different single-DB trajectory groups might be attributable, at least in part, to a subgroup of children who show comorbidity across different DBs. The best example of this is father’s adolescent CP, a risk factor common to all three single-DB high trajectories, but only related to the HI + NC + PA joint-DBs high trajectory group. It is also worth mentioning that the identified risk factors and the strength of their associations with preschool single-DB high trajectories are consistent with those reported for similar early risk factors linked with preschool trajectories of HI [24,25,27,30], NC [26,29], and PA [23,28,32,33] or with general externalizing problems [34,35,36]. This consistency across studies lends further support to the present findings.

### 4.1. Clinical Implications for Child Healthcare

The refinement of clinical profiles is increasingly advocated to address the needs of children with DBs and guide prevention and treatment interventions [15,52]. The results of the present study support the idea that this process should start during early childhood with the investigation of multiple DBs and their comorbidity rather than single-DB categories [3,53]. The mere comparison between the average proportion of risk factors specific to one single-DB high trajectory or another and the same proportion for joint-DBs high trajectory groups suggest that there is more specificity and less commonality in the risk factors experienced by children within each DB than there is between DB categories. Taking into account the comorbidity between different DBs is, thus, essential to identify the specific needs of young children and their families. A more individual, person-centered perspective across DBs may be needed to improve our ability to effectively prevent or treat them [54,55].

The comparison of the comorbid joint-DBs trajectories of HI + NC and NC + PA illustrates the relevance of such perspective. Children from these trajectory groups have in common a high trajectory of NC across preschool years and a mother with conduct problems in early adulthood. However, children of the first group also followed a high trajectory of HI, whereas children of the second group also followed a high trajectory of PA; children in the first group were more likely to be males and to have a difficult temperament, whereas children in the second group were of either sex and were not rated as difficult by their parents; children in the first group had few or no siblings and were more likely to live in poverty, whereas children in the second group had more siblings than average and lived in a family with a better than average chance to be above the poverty level; children in the first group had a mother who experienced depressive symptoms and a father of average age, whereas children in the second group had a younger father than average and a mother who did not suffer from depression, and, unlike families of the first group, families of the children in the second group had a higher level of dysfunction and conflicts, and fathers were more likely to adopt coercive behaviors. To say the least, the children and their families in these two groups seem quite different, although they were all included in the single-DB trajectory group of high-NC.

From a clinical perspective, the results of this study are important because they suggest that different longitudinal profiles across DBs observable early in children’s development may be associated with different familial and environmental characteristics as well. It is, therefore, possible that assessing early the manifestations of multiple DBs may be informative on the nature of the risk factors potentially involved, which could be investigated in the child’s family and social environment. If validated, these risk factors may then become targets for preventive intervention for the benefit of the child. For example, children exhibiting HI behaviors and other DBs would be likely to have a mother or father with antisocial behaviors, whereas a child showing only HI problems would, on the other hand, be likely to live with both parents struggling with depressive symptoms. After confirming these suspicions, support for parents of these different children exhibiting early HI behaviors would partly aim at different targets through differentiated interventions fostering parental emotional wellbeing or behavioral control and related parenting practices with young children. More refined profiles across DBs and their differential associations with early risk factors may also guide the investigation of etiological mechanisms, which could, in turn, result in the refinement of theoretical models and the development of new tailored interventions. Risk factors that cannot be acted upon in a child’s early years, such as mother smoking during pregnancy, may still be useful in that fashion.

In the present study, children who followed a high trajectory for both HI and PA were all part of the same joint-DBs trajectory group, which followed high trajectories for all three DBs. The fact that they were also highly non-compliant throughout preschool years is consistent with reports of more impaired functioning in children with comorbid attention deficit–hyperactivity and behavior problems [56,57]. Their least-adjusted profile in first grade among children of all trajectory groups, based on teachers’ assessment of their behavior and school performance [1], also closely matches the associations reported in older children [6]. Hence, this specific subgroup of children—the most prevalent among joint trajectory groups including a high trajectory for one DB or another—could be identified very early in their development, and significant risk factors could be observed in the prenatal to age 5-month period.

Interestingly, the risk factors specific to this trajectory group, i.e., father’s adolescent conduct problems (for its potential intergenerational transmission [14]) and mother smoking during pregnancy (for its potential influence on fetal development and on epigenetic mechanisms in utero [7]), are both coherent as potential etiological factors of an early, genetically predisposed comorbid condition. However, the fact that these risk factors were common to different categories of DBs in several previous studies incited authors to conclude that they constituted general risk factors that were not discriminatory among DBs, whereas the present results suggest that they may actually be specific to the comorbid condition favoring the simultaneous development of high levels for all three forms of DBs. In that case, it is the presence of HI + NC + PA children in single-DB trajectory groups of high-HI, high-NC, or high-PA that gives the impression of common factors to these different categories of DBs.

This has obvious implications for screening and targeting these children for preventive intervention, as they are most at risk of pursuing an antisocial trajectory into adulthood [14,17]. The longitudinal follow-up of this comorbid pattern (and others) may clarify the role of environmental risk factors from the prenatal period onwards. It may also help clarify the issue of the relative homotypic and heterotypic continuity observed in distinct DBs from preschool to adolescence [2], as our findings suggest that each DB is a mosaic of a minority of ‘pure’ or monomorbid cases, and a majority of children with comorbid patterns associated to a large extent with specific risk factors.

### 4.2. Strengths and Limitations

To our knowledge, this study is the first to examine early risk factors associated with three distinct categories of single DBs and to compare them with risk factors linked with joint trajectories across these three types of DBs over the preschool years in the same sample. A large population cohort and longitudinal phenotypes to characterize DBs are important strengths of the present study. Using trajectories of DBs provided indicators of a child’s level for each DB’s developmental course and a dynamic point of comparison with peers. This strategy avoided the caveats of cross-sectional assessments in a developmental period marked by maturational changes in the children’s behaviors [14,58]. Finally, the use of a large sample to compare the risk factors for individual single DBs and monomorbid and comorbid joint trajectories across DBs is an important asset, given that such comparisons are usually made between studies of different populations, including different sets and measures of risk factors.

Nevertheless, our study is not without limitations. First, relying only on the mother as the informant for early DBs was not optimal. However, as in most large-scale studies at that age period, an affordable alternative reporting source for such a large sample was not available [9,31]. Second, despite the advantage provided by the trajectories, these constructs required the examination of the same behaviors across different time points and, thus, could not capture potential developmentally based distinctions among children based on unaccounted behaviors. Third, the categories of DBs examined were not standard diagnostic categories, although they corresponded to symptoms of each DSM disruptive behavior disorder (i.e., ADHD, ODD, and CD [59]). Given the requirements of the trajectory method, there were obvious constraints in regard to which behaviors could be reasonably and meaningfully assessed in children from age 1½ to 5 years. On a related vein, it is important to recall that our analyses did not compare children who score below a predetermined threshold with those who exceed it. Rather, the present results are based on empirically derived longitudinal models of DBs and compare children with higher levels to their peers with moderate or low levels for all DBs. Although this strategy has proven valid for distinguishing children based on various risk factors as well as early or later outcomes in previous studies [1,17,23,24,25,31,32,33,36], the results must be interpreted in the context of the method used. In any case, beyond the actual categories of DBs studied, it is the potential patterns of comorbidity among these behaviors within individuals that need more attention. Fourth, despite being widely used, group-based trajectory modeling (also called latent class growth analysis) has been criticized for providing results that are not easily replicated across studies or samples [60]. However, it has been suggested that variations in the methodology used across different studies—e.g., criteria and indicators for model selection and group classification or disparity in samples—likely account for this difficulty in replication [60]. In the present study, we followed the recommendations for conducting and reporting on such methodology [61,62]. Importantly, the trajectories identified for each DB were similar to those reported in studies of single DBs with preschool children [23,25,28,29,30,31]. Finally, as the present study was based on a 1997–1998 population birth cohort of North American children raised mainly in a French-speaking culture, replications are necessary to determine the generalizability of the results.

## 5. Conclusions

The above limitations notwithstanding, the results of the present study examining longitudinal risk factors associated with different types of DBs and with their comorbidity patterns support investigating comorbidity as an important strategy to (a) develop a refined and evidence-based phenomenology of preschool DBs, (b) improve our understanding of the etiological processes leading to these distinct but related behaviors and their inter-related patterns from early childhood onwards, and (c) plan for early tailored preventive or curative interventions. Clinical research reports have emphasized, for almost three decades, the importance of looking at comorbidities whenever a child is referred for treatment [52,53,63]. Longitudinal follow-ups of population cohorts from early childhood onwards have also concluded that subtypes of DBs should not be aggregated in young children because they have different developmental trajectories and require specific corrective interventions [10]. Yet, comorbidity among early childhood disorders still remains overlooked [64,65]. The current results emphasize that studies should move beyond the investigation of a single DB to refine the definition of DBs, guide the study of etiological mechanisms and risk assessment, and develop intervention strategies tailored to the specific needs of children and their families.

## Figures and Tables

**Table 1:** Predictors of single-DB high trajectory groups of HI, NC, or PA over preschool years (OR ^1^): Full model.

Predictors ^2^	Single-DB High Trajectory		
	HI (N = 305)	NC (N = 388)	PA (N = 321)
Parents’ pre-conception characteristics			
Mother’s adolescent conduct problems	1.10 [0.97, 1.25]	1.08 [0.96, 1.22]	1.08 [0.96, 1.23]
Father’s adolescent conduct problems	**1.16 [1.01, 1.33]**	**1.17 [1.04, 1.32]**	1.12 [0.97, 1.28]
Mother/No High school Diploma	0.97 [0.68, 1.39]	0.95 [0.69, 1.36]	1.14 [0.80, 1.61]
Father/No High school Diploma	1.20 [0.86, 1.66]	1.19 [0.88, 1.60]	1.25 [0.91, 1.72]
Mother’s age at birth of first child	0.86 [0.69, 1.07]	0.96 [0.82, 1.12]	0.95 [0.80, 1.12]
Pregnancy characteristics: Mother			
Smoking during pregnancy (y/n)	**1.44 [1.05, 1.98]**	1.21 [0.90, 1.61]	**1.40 [1.03, 1.90]**
Alcohol during pregnancy (y/n)	0.93 [0.66, 1.29]	1.02 [0.76, 1.36]	1.01 [0.73, 1.39]
Drugs during pregnancy (y/n)	0.83 [0.26, 2.64]	1.14 [0.43, 3.04]	1.03 [0.32, 3.24]
Perinatal characteristics			
Mother’s age at child’s birth	0.84 [0.69, 1.02]	**0.82 [0.69, 0.98]**	0.86 [0.71, 1.04]
Father’s age at child’s birth	1.08 [0.95, 1.23]	1.00 [0.87, 1.14]	0.92 [0.77, 1.09]
Premature or Low Birthweight (y/n)	**1.81 [1.12, 2.92]**	1.29 [0.81, 2.08]	1.39 [0.83, 2.33]
Male gender	**2.68 [2.02, 3.57]**	**1.84 [1.44, 2.35]**	**2.61 [1.98, 3.45]**
Number of siblings	0.98 [0.83, 1.16]	1.05 [0.91, 1.21]	**1.44 [1.24, 1.68]**
Age 5 months: Child & Family Characteristics			
Child’s Difficult Temperament	1.04 [0.90, 1.20]	1.02 [0.89, 1.16]	1.06 [0.91, 1.22]
Non-intact family	**1.48 [1.03, 2.13]**	1.28 [0.91, 1.79]	0.97 [0.68, 1.39]
Family dysfunction	0.95 [0.81, 1.12]	1.07 [0.93, 1.23]	1.13 [0.97, 1.31]
Income < Poverty level (y/n)	1.09 [0.77, 1.54]	1.03 [0.74, 1.41]	0.94 [0.67, 1.33]
Mother’s Depression Symptoms	**1.35 [1.16, 1.56]**	**1.23 [1.08, 1.41]**	**1.20 [1.04, 1.39]**
Father’s Depression Symptoms	**1.17 [1.01, 1.35]**	1.08 [0.95, 1.23]	1.12 [0.97, 1.29]
Mother’s adult conduct problems	1.07 [0.93, 1.22]	**1.13 [1.01, 1.27]**	**1.15 [1.01, 1.31]**
Father’s adult conduct problems	0.98 [0.85, 1.14]	1.13 [1.01, 1.27]	1.02 [0.89, 1.17]
Mother’s frequency of alcohol use	0.97 [0.81, 1.17]	1.03 [0.88, 1.21]	0.97 [0.82, 1.15]
Father’s frequency of alcohol use	0.90 [0.77, 1.05]	0.95 [0.82, 1.09]	0.98 [0.84, 1.13]
Parents’ drug use (y/n)	1.02 [0.89, 1.17]	1.01 [0.89, 1.13]	0.97 [0.84, 1.12]
Age 5 months: Parenting			
Self-efficiency Mother	0.92 [0.78, 1.10]	0.92 [0.79, 1.08]	0.93 [0.80, 1.09]
Coercion Mother	1.09 [.95, 1.26]	1.11 [0.98, 1.26]	1.07 [0.92, 1.23]
Affection/pleasure Mother	1.02 [0.87, 1.21]	1.01 [0.87, 1.16]	1.00 [0.86, 1.17]
Overprotection Mother	1.14 [0.98, 1.33]	0.98 [0.86, 1.12]	0.93 [0.81, 1.08]
Positive mother-child interactions	0.93 [0.80, 1.08]	0.96 [0.84, 1.09]	0.93 [0.81, 1.06]
Coercion Father	1.11 [0.96, 1.29]	1.11 [0.98, 1.27]	0.99 [0.85, 1.16]
Affection/pleasure Father	1.06 [0.91, 1.24]	0.97 [0.86, 1.10]	0.97 [0.85, 1.11]

^1^: Odd Ratios (OR) and 95% CI. HI: hyperactivity–impulsivity; NC: non-compliance; PA: physical aggression. Statistically significant predictors are indicated in bold. ^2^: Continuous predictors unless specified otherwise.

**Table 2:** Predictors of single-DB high trajectory groups of HI, NC, or PA over preschool years (OR ^1^): Final model.

Predictors ^2^	Single-DB High Trajectory		
	HI (N = 305)	NC (N = 388)	PA (N = 321)
Parents’ pre-conception characteristics			
Father’s adolescent conduct problems	1.19 [1.05, 1.36]	1.20 [1.07, 1.34]	1.15 [1.02, 1.31]
Pregnancy characteristics: Mother			
Smoking during pregnancy (y/n)	1.53 [1.14, 2.06]		1.49 [1.12, 1.99]
Perinatal characteristics			
Mother’s age at child’s birth	0.79 [0.69, 0.91]	0.81 [0.72, 0.92]	0.78 [0.67, 0.90]
Premature or Low Birthweight (y/n)	1.83 [1.14, 2.93]		
Male gender	2.76 [2.08, 3.66]	1.86 [1.46, 2.36]	2.56 [1.95, 3.35]
Number of siblings			1.50 [1.31, 1.70]
Age 5 months: Child & Family Characteristics			
Non-intact family	1.62 [1.18, 2.24]	1.46 [1.08, 1.97]	
Family dysfunction			1.18 [1.02, 1.35]
Mother’s Depression Symptoms	1.36 [1.19, 1.55]	1.32 [1.18, 1.48]	1.25 [1.09, 1.43]
Father’s Depression Symptoms	1.19 [1.04, 1.36]		
Mother’s adult conduct problems		1.17 [1.05, 1.31]	1.18 [1.05, 1.33]
Age 5 months: Parenting			
Coercion Mother	1.17 [1.03, 1.33]	1.14 [1.01, 1.28]	
Overprotection Mother	1.16 [1.01, 1.34]		
Coercion Father		1.15 [1.02, 1.29]	
Model fitting			
AIC (ΔAIC)	1453.979 (−26.125)	1952.845 (−32.633)	1616.509 (−27.768)
BIC (ΔBIC)	1515.523 (−143.617)	2003.199 (−161.315)	1666.863 (−156.45)

^1^: Odd Ratios (OR) and 95% CI. ^2^: Continuous predictors unless specified otherwise. HI: hyperactivity–impulsivity; NC: non-compliance; PA: physical aggression. Model’s Akaike information criterion (AIC) and Bayesian information criterion (BIC); ΔAIC and ΔBIC comparing final to full models’ fit.

**Table 3:** Predictors of joint-DBs high trajectory groups across HI, NC, and PA during preschool Years (OR ^1^): Full model.

Predictors ^2^	Disruptive Behaviors Joint-Trajectories					
	Monomorbid Joint Trajectory-Groups			Comorbid Joint Trajectory-Groups		
	HI_only_ (N = 67; 22.0 ^3^)	NC_only_ (N = 93; 23.8 ^4^)	PA_only_ (N = 123; 38.2 ^5^)	HI + NC (N = 97; 31.8 ^3^/24.9 ^4^)	NC + PA (N = 57; 14.8 ^4^/17.8 ^5^)	HI + NC + PA (N = 141; 46.2 ^3^/36.5 ^4^/44.0 ^5^)
Parents’ pre-conception characteristics						
Mother’s adolescent conduct problems	1.09 [0.85, 1.40]	1.08 [0.88, 1.34]	1.08 [0.90, 1.31]	1.12 [0.90, 1.39]	1.02 [0.77, 1.35]	1.06 [0.89, 1.25]
Father’s adolescent conduct problems	1.05 [0.79, 1.40]	1.13 [0.91, 1.41]	0.97 [0.78, 1.20]	1.11 [0.88, 1.39]	1.15 [0.87, 1.53]	**1.25 [1.04, 1.50]**
Mother/No High school Diploma	1.56 [0.80, 3.03]	1.29 [0.71, 2.36]	1.12 [0.67, 1.87]	0.44 [0.22, 0.86]	1.01 [0.46, 2.20]	1.23 [0.76, 1.97]
Father/No High school Diploma	1.02 [0.54, 1.96]	0.81 [0.44, 1.49]	1.16 [0.71, 1.89]	1.37 [0.80, 2.35]	1.71 [0.86, 3.43]	1.24 [0.79, 1.93]
Mother’s age at birth of first child	0.55 [0.22, 1.37]	1.09 [0.89, 1.33]	0.99 [0.80, 1.24]	0.76 [0.43, 1.35]	0.76 [0.37, 1.56]	0.94 [0.75, 1.18]
Pregnancy characteristics: Mother						
Smoking during pregnancy (y/n)	1.10 [0.58, 2.07]	0.79 [0.44, 1.41]	1.39 [0.87, 2.21]	1.26 [0.74, 2.14]	0.96 [0.47, 1.97]	**1.69 [1.10, 2.60]**
Alcohol during pregnancy (y/n)	0.81 [0.41, 1.61]	1.14 [0.69, 1.90]	0.80 [0.49, 1.31]	0.78 [0.44, 1.37]	1.17 [0.59, 2.30]	1.11 [0.70, 1.77]
Drugs during pregnancy (y/n)	1.12 [0.42, 3.00]	1.89 [0.37, 9.71]	1.87 [0.34, 10.42]	1.32 [0.25, 7.11]	4.5 [0.44, 46.63]	0.72 [0.17, 3.03]
Perinatal characteristics						
Mother’s age at child’s birth	1.06 [0.64, 1.78]	0.78 [0.55, 1.10]	0.95 [0.73, 1.25]	1.03 [0.71, 1.51]	1.27 [0.76, 2.14]	0.76 [0.58, 1.00]
Father’s age at child’s birth	1.12 [0.86, 1.44]	0.83 [0.57, 1.22]	0.96 [0.75, 1.22]	1.15 [0.97, 1.36]	0.56 [0.30, 1.05]	0.95 [0.75, 1.21]
Premature or Low Birthweight (y/n)	2.26 [0.98, 5.17]	1.07 [0.42, 2.71]	1.08 [0.49, 2.40]	1.50 [0.67, 3.36]	1.00 [0.28, 3.51]	1.70 [0.85, 3.40]
Male gender	**2.31 [1.32, 4.03]**	1.09 [0.70, 1.70]	**2.65 [1.75, 4.02]**	**1.83 [1.17, 2.88]**	1.12 [0.63, 1.99]	**3.79 [2.47, 5.80]**
Number of siblings	0.86 [0.59, 1.26]	0.99 [0.75, 1.31]	**1.53 [1.24, 1.89]**	**0.56 [0.39, 0.80]**	1.38 [0.98, 1.95]	**1.32 [1.06, 1.64]**
Age 5 months: Child & Family Characteristics						
Child’s Difficult Temperament	0.98 [0.74, 1.30]	0.90 [0.70, 1.15]	1.15 [0.93, 1.43]	1.20 [0.96, 1.51]	1.06 [0.78, 1.44]	0.93 [0.76, 1.15]
Non-intact family	1.39 [0.69, 2.84]	0.78 [0.38, 1.60]	0.56 [0.31, 0.99]	1.60 [0.86, 2.99]	1.25 [0.58, 2.71]	1.36 [0.83, 2.21]
Family dysfunction	0.71 [0.51, 0.98]	1.00 [0.78, 1.29]	**1.26 [1.02, 1.57]**	1.08 [0.84, 1.39]	**1.39 [1.02, 1.91]**	0.94 [0.75, 1.16]
Income < Poverty level (y/n)	0.71 [0.35, 1.45]	0.92 [0.48, 1.74]	1.00 [0.60, 1.68]	1.45 [0.83, 2.56]	**0.38 [0.16, 0.94]**	1.02 [0.63, 1.64]
Mother’s Depression Symptoms	**1.44 [1.11, 1.87]**	1.16 [0.91, 1.49]	1.02 [0.81, 1.27]	1.13 [0.89, 1.44]	0.98 [0.70, 1.36]	**1.44 [1.17, 1.76]**
Father’s Depression Symptoms	**1.47 [1.15, 1.88]**	1.10 [0.88, 1.38]	1.20 [0.97, 1.47]	1.09 [0.87, 1.37]	1.14 [0.86, 1.53]	1.07 [0.86, 1.32]
Mother’s adult conduct problems	0.94 [0.69, 1.28]	1.07 [0.86, 1.33]	**1.20 [1.01, 1.45]**	1.16 [0.95, 1.42]	1.22 [0.94, 1.58]	1.09 [0.90, 1.31]
Father’s adult conduct problems	1.26 [0.98, 1.62]	1.05 [0.82, 1.34]	1.20 [1.00, 1.44]	0.96 [0.75, 1.22]	1.11 [0.84, 1.47]	0.88 [0.72, 1.09]
Mother’s frequency of alcohol use	0.92 [0.64, 1.32]	1.16 [0.89, 1.52]	0.97 [0.75, 1.25]	0.95 [0.69, 1.29]	0.93 [0.65, 1.34]	0.98 [0.76, 1.27]
Father’s frequency of alcohol use	1.03 [0.76, 1.39]	1.11 [0.85, 1.43]	1.09 [0.88, 1.35]	0.91 [0.70, 1.19]	1.06 [0.77, 1.46]	0.88 [0.71, 1.10]
Parents’ drug use	0.78 [0.54, 1.15]	0.99 [0.80, 1.24]	0.81 [0.61, 1.08]	1.00 [0.80, 1.24]	0.67 [0.42, 1.07]	1.11 [0.93, 1.32]
Age 5 months: Parenting						
Self-efficiency Mother	0.91 [0.67, 1.24]	0.83 [0.63, 1.08]	0.86 [0.69, 1.08]	0.87 [0.66, 1.14]	1.11 [0.76, 1.62]	0.98 [0.77, 1.25]
Coercion Mother	1.19 [0.91, 1.55]	1.22 [0.99, 1.50]	1.06 [0.86, 1.31]	1.06 [0.84, 1.33]	1.10 [0.83, 1.47]	1.06 [0.87, 1.29]
Affection/pleasure Mother	1.13 [0.76, 1.67]	1.01 [0.79, 1.28]	1.02 [0.83, 1.25]	1.02 [0.79, 1.33]	1.00 [0.74, 1.36]	0.98 [0.79, 1.21]
Overprotection Mother	**1.38 [1.01, 1.87]**	0.88 [0.68, 1.13]	0.96 [0.77, 1.19]	1.27 [0.98, 1.63]	0.85 [0.62, 1.18]	0.96 [0.78, 1.19]
Positive mother-child interactions	0.87 [0.66, 1.16]	1.00 [0.78, 1.28]	0.94 [0.78, 1.14]	1.00 [0.78, 1.30]	1.01 [0.75, 1.38]	0.90 [0.74, 1.10]
Coercion Father	1.16 [0.87, 1.55]	1.01 [0.79, 1.28]	0.77 [0.59, 1.00]	1.19 [0.95, 1.50]	**1.33 [1.01, 1.75]**	1.07 [0.86, 1.34]
Affection/pleasure Father	1.20 [0.86, 1.67]	1.02 [0.80, 1.29]	1.03 [0.85, 1.25]	1.08 [0.83, 1.41]	0.80 [0.63, 1.00]	1.00 [0.82, 1.21]

^1^: Odd Ratios (OR) and 95% CI. Significant predictors are indicated in **bold**. ^2^: Continuous predictors unless specified otherwise. HI: Hyperactivity-Impulsivity; NC: Non-Compliance; PA: Physical Aggression. ^3,4,5^: % of sample’s High-HI ^3^, High-NC ^4^, and High-PA ^5^ children in joint-DBs trajectory group.

**Table 4:** Predictors of joint-DBs high trajectory groups across HI, NC, and PA during preschool Years (OR ^1^): Final model.

Predictors ^2^	Disruptive Behaviors Joint-Trajectories					
	Monomorbid Joint Trajectory-Groups			Comorbid Joint Trajectory-Groups		
	HI_only_ (N = 67; 22.0 ^3^)	NC_only_ (N = 93; 23.8 ^4^)	PA_only_ (N = 123; 38.2 ^5^)	HI + NC (N = 97; 31.8 ^3^/24.9 ^4^)	NC + PA (N = 57; 14.8 ^4^/17.8 ^5^)	HI + NC + PA (N = 141; 46.2 ^3^/36.5 ^4^/44.0 ^5^)
Parents’ pre-conception characteristics						
Father’s adolescent conduct problems						1.24 [1.05, 1.47]
Mother’s age at birth of first child	0.56 [0.34, 0.91]					
Pregnancy characteristics: Mother						
Smoking during pregnancy (y/n)						1.94 [1.31, 2.89]
Perinatal characteristics						
Mother’s age at child’s birth		0.74 [0.58, 0.93]				0.65 [0.52, 0.81]
Father’s age at child’s birth					0.57 [0.36, 0.92]	
Premature or Low Birthweight (y/n)	2.55 [1.17, 5.55]					
Male gender	2.31 [1.35, 3.96]		2.62 [1.75, 3.93]	1.78 [1.15, 2.76]		3.71 [2.45, 5.62]
Number of siblings			1.43 [1.21, 1.68]	0.61 [0.46, 0.80]	1.63 [1.25, 2.14]	1.46 [1.21, 1.76]
Age 5 months: Child & Family Characteristics						
Child’s Difficult Temperament				1.34 [1.09, 1.64]		
Family dysfunction			1.37 [1.15, 1.63]		1.45 [1.23, 1.87]	
Income < Poverty level (y/n)				1.97 [1.23, 3.17]	0.44 [0.20, 0.96]	
Mother’s Depression Symptoms	1.33 [1.06, 1.66]			1.28 [1.05, 1.56]		1.48 [1.26, 1.74]
Father’s Depression Symptoms	1.40 [1.13, 1.73]					
Mother’s adult conduct problems			1.21 [1.03, 1.43]	1.25 [1.05, 1.50]	1.30 [1.04, 1.63]	
Father’s adult conduct problems			1.20 [1.02, 1.40]			
Mother’s frequency of alcohol use		1.27 [1.03, 1.57]				
Age 5 months: Parenting						
Coercion Mother		1.34 [1.12, 1.60]				
Overprotection Mother	1.41 [1.08, 1.83]					
Coercion Father					1.41 [1.13, 1.77]	
Model fitting						
AIC (ΔAIC)	484.693 (−31.080)	675.090 (−38.422)	793.693 (−28.991)	653.048 (−23.759)	447.776 (−33.695)	810.038 (−32.956)
BIC (ΔBIC)	521.849 (−163.776)	693.393 (−187.545)	825.769 (−167.987)	690.338 (−156.937)	484.889 (−166.240)	847.518 (−166.815)

^1^: Odd Ratios (OR) and 95% CI. ^2^: Continuous predictors unless specified otherwise. HI: Hyperactivity-Impulsivity; NC: Non-Compliance; PA: Physical Aggression. ^3,4,5^: % of sample’s High-HI ^3^, High-NC ^4^, and High-PA ^5^ children in joint-DBs trajectory group. Model’s Akaike Information Criterion (AIC) and Bayesian Information Criterion (BIC); ΔAIC and ΔBIC comparing final to full models’ fit.

## Data Availability

Data used in the present study are owned by Gouvernement du Québec and are under the responsibility of Institut de la statistique du Québec. Data cannot be shared publicly and are protected by the ACCESS TO INFORMATION ACT (Québec), as indicated in the consent form signed by the participants and approved by the Ethics Committee of Santé Québec Division. Data are available from the Centre d’accès aux données de recherche de l’Institut de la Statistique du Québec (CADRISQ) for researchers who meet the criteria for access to confidential data. Please contact Nancy Illick: nancy.illick@stat.gouv.qc.ca. See also: http://www.jesuisjeserai.stat.gouv.qc.ca/informations_chercheurs/acces_an.html.

## Abbreviations

DBs	Disruptive behaviors.
HI	Hyperactivity–impulsivity.
NC	Non-compliance.
PA	Physical aggression.
Trajectory (low, moderate, or high)	A course of behavior from age 1½ to 5 years.
Trajectory group	Children following a similar trajectory.
Single DB	One type of DB (i.e., HI, NC, or PA) examined separately.
Single-DB high trajectory	A high trajectory for a single DB (e.g., high-HI, high-NC, or high-PA).
Single-DB high trajectory group	Children following a high trajectory for a single DB.
Joint DBs	Refers to the three DBs (HI, NC, and PA) examined jointly.
Joint-DBs high trajectory	A high trajectory for at least one DB when the three DBs are examined jointly.
Joint-DBs high trajectory group	Children following a joint-DBs high trajectory.
Monomorbid joint-DBs high trajectory group (i.e., high-HI_only_, high-NC_only_, high-PA_only_)	Children following a joint-DBs high trajectory for only one DB when the three DBs are examined jointly; trajectories followed for the other two DBs are either low or moderate.
Comorbid joint-DBs high trajectory group (i.e., high-HI + NC, high-NC + PA or high-HI + NC + PA)	Children following a joint-DBs high trajectory for more than one DB when the three DBs are examined jointly; in joint-DBs high trajectory groups including two high DBs, the trajectory followed for the remaining third DB is either low or moderate.
Reference group	Children following either low or moderate trajectories (i.e., no high trajectory).
